# Factors Predicting Oncological Outcomes of Radical Nephroureterectomy for Upper Tract Urothelial Carcinoma in Taiwan

**DOI:** 10.3389/fonc.2021.766576

**Published:** 2022-01-13

**Authors:** I-Hsuan Alan Chen, Chao-Hsiang Chang, Chi-Ping Huang, Wen-Jeng Wu, Ching-Chia Li, Chung-Hsin Chen, Chao-Yuan Huang, Chi-Wen Lo, Chih-Chin Yu, Chung-You Tsai, Wei-Che Wu, Jen-Shu Tseng, Wun-Rong Lin, Yuan-Hong Jiang, Yu-Khun Lee, Yeong-Chin Jou, Ian-Seng Cheong, Thomas Y. Hsueh, Allen W. Chiu, Yung-Tai Chen, Jih-Sheng Chen, Bing-Juin Chiang, Yao-Chou Tsai, Wei Yu Lin, Chia-Chang Wu, Jen-Tai Lin, Chia-Cheng Yu

**Affiliations:** ^1^ Division of Urology, Department of Surgery, Kaohsiung Veterans General Hospital, Kaohsiung, Taiwan; ^2^ Institute of Clinical Medicine, School of Medicine, National Yang Ming Chiao Tung University, Taipei, Taiwan; ^3^ Division of Urology, Department of Surgery, Tri-Service General Hospital, National Defense Medical Center, Taipei, Taiwan; ^4^ Department of Urology, China Medical University and Hospital, Taichung, Taiwan; ^5^ School of Medicine, China Medical University, Taichung, Taiwan; ^6^ Department of Urology, Kaohsiung Medical University Hospital, Kaohsiung, Taiwan; ^7^ Department of Urology, School of Medicine, College of Medicine, Kaohsiung Medical University, Kaohsiung, Taiwan; ^8^ Graduate Institute of Clinical Medicine, College of Medicine, Kaohsiung Medical University, Kaohsiung, Taiwan; ^9^ Department of Urology, National Taiwan University Hospital, College of Medicine, National Taiwan University, Taipei, Taiwan; ^10^ Division of Urology, Department of Surgery, Taipei Tzu Chi Hospital, The Buddhist Tzu Chi Medical Foundation, New Taipei, Taiwan; ^11^ School of Medicine, Buddhist Tzu Chi University, Hualien, Taiwan; ^12^ Division of Urology, Department of Surgery, Far Eastern Memorial Hospital, New Taipei, Taiwan; ^13^ Department of Healthcare Information and Management, Ming Chuan University, Taipei, Taiwan; ^14^ Institute of Biomedical Engineering, National Taiwan University, Taipei, Taiwan; ^15^ Department of Urology, MacKay Memorial Hospital, Taipei, Taiwan; ^16^ Department of Medicine, Mackay Medical College, Taipei, Taiwan; ^17^ Department of Urology, Hualien Tzu Chi Hospital, Buddhist Tzu Chi Medical Foundation and Tzu Chi University, Hualien, Taiwan; ^18^ Department of Urology, Ditmanson Medical Foundation Chiayi Christian Hospital, Chiayi, Taiwan; ^19^ Department of Health and Nutrition Biotechnology, Asian University, Taichung, Taiwan; ^20^ Division of Urology, Department of Surgery, Taipei City Hospital Renai Branch, Taipei, Taiwan; ^21^ Department of Urology, School of Medicine, National Yang Ming Chiao Tung University, Taipei, Taiwan; ^22^ College of Medicine, National Yang Ming Chiao Tung University, Taipei, Taiwan; ^23^ Department of Urology, Taiwan Adventist Hospital, Taipei, Taiwan; ^24^ College of Medicine, Fu-Jen Catholic University, New Taipei City, Taiwan; ^25^ Department of Urology, Cardinal Tien Hospital, New Taipei City, Taiwan; ^26^ Department of Life Science, College of Science, National Taiwan Normal University, Taipei, Taiwan; ^27^ Department of Urology, School of Medicine, College of Medicine, Taipei Medical University, Taipei, Taiwan; ^28^ Department of Urology, Taipei Medical University Hospital, Taipei Medical University, Taipei, Taiwan; ^29^ Division of Urology, Department of Surgery, Chang Gung Memorial Hospital, Chia-Yi, Taiwan; ^30^ Chang Gung University of Science and Technology, Chia-Yi, Taiwan; ^31^ Department of Medicine, Chang Gung University, Taoyuan, Taiwan; ^32^ Department of Urology, Shuang Ho Hospital, Taipei Medical University, New Taipei, Taiwan; ^33^ TMU Research Center of Urology and Kidney, Taipei Medical University, Taipei, Taiwan

**Keywords:** kidney pelvis, nephroureterectomy, risk factors, survival, ureter, urinary bladder, urinary tract, urothelial carcinoma

## Abstract

**Background:**

Taiwan is one of the endemic regions where upper tract urothelial carcinoma (UTUC) accounts for approximately a third of all urothelial tumors. Owing to its high prevalence, extensive experience has been accumulated in minimally invasive radical nephroureterectomy (RNU). Although a variety of predictive factors have been explored in numerous studies, most of them were on a single-center or limited institutional basis and data from a domestic cohort are lacking.

**Objective:**

This study aims to identify significant predicting factors of oncological outcomes, including overall survival (OS), cancer-specific survival (CSS), disease-free survival (DFS), and intravesical recurrence-free survival (IVRFS), following RNU for UTUC in Taiwan.

**Methods:**

A multicenter registry database, Taiwan UTUC Collaboration Group, was utilized to analyze oncological outcomes of 3,333 patients undergoing RNU from 1988 to 2021 among various hospitals in Taiwan. Clinicopathological parameters were recorded according to the principles established by consensus meetings. The Kaplan-Meier estimator was utilized to estimate the survival rates, and the curves were compared using the stratified log-rank test. Univariate and multivariate analyses were performed with the Cox proportional hazard model to explore potential predicting factors.

**Results:**

With a median follow-up of 41.8 months in 1,808 patients with complete information, the 5-year IVRFS, DFS, CSS, and OS probabilities were 66%, 72%, 81%, and 70%, respectively. In total, 482 patients experienced intravesical recurrence, 307 died of UTUC, and 583 died of any cause. Gender predominance was female (57%). A total of 1,531 patients (84.7%) had high-grade tumors; preoperative hydronephrosis presented in 1,094 patients (60.5%). Synchronous bladder UC was identified in 292 patients (16.2%). Minimally invasive procedures accounted for 78.8% of all surgeries, including 768 hand-assisted laparoscopic (42.5%) and 494 laparoscopic (27.3%) approaches. Synchronous bladder UC was the dominant adverse predicting factor for all survival outcomes. Other independent predicting factors for OS, CSS, and DFS included age ≧70, presence of preoperative hydronephrosis, positive surgical margin, LVI, pathological T and N staging, and laparoscopic RNU.

**Conclusion:**

Synchronous UC of the urinary bladder is an independent adverse prognostic factor for survival in UTUC. The presence of preoperative hydronephrosis was also corroborated as a disadvantageous prognostic factor. Our multivariate analysis suggested that laparoscopic RNU might provide better oncological control.

## Introduction

Upper tract urothelial carcinoma (UTUC) comprises approximately 5% to 10% of all urothelial cancer (1). Taiwan is one of the endemic regions where UTUC accounts for 30% of all urothelial tumors (2). With the estimated annual incidence of up to 2 new cases per 100,000 person-years in the Western countries, the Taiwan Cancer Registry Annual Report depicted the age-standardized incidence rate of UTUC was 3.71 in men and 3.99 in women per 100,000 population in 2018. Radical nephroureterectomy (RNU) is the standard primary treatment for localized or even locally advanced UTUC. Owing to its high prevalence in Taiwan, apart from conventional open RNU, extensive experience was obtained in minimally invasive surgical approaches for managing UTUC.

On account of its relatively low incidence across the world, focused collaborative efforts are required to better understand the behavior of UTUC. Globally, a number of multi-institutional database have contributed to the prediction of prognosis and therapeutic responses following RNU (3), but the sample size was limited and interethnic variations and regional differences might exist in these cohorts. In order to obtain comprehensive information about the prognosis locally, a multicenter registry database, the Taiwan UTUC Collaboration Group, was established to record clinical data and treatment outcomes of patients who underwent RNU from 1988 to 2021 among various hospitals in Taiwan. In contrast with the National Health Insurance Research Database (NHIRD) of Taiwan, our dataset could provide detailed clinical information and mitigate the effects of unmeasured confounders. Additionally, diagnosis validity would be confirmed after serial consensus meetings. Robust results might be expected through collaborative work among medical centers and regional hospitals.

A variety of predictive factors have been explored in numerous studies, including patient, tumor, and pathological factors, to forecast outcomes of patients with UTUC (4). Gender (5), preoperative blood-based biomarkers (6, 7), tumor stage (8), and location (9) had been identified as pivotal predictive factors for UTUC following RNU in a Taiwanese population. Nevertheless, most results were derived from single-center or limited institutional studies, and data from a domestic cohort are lacking. The aim of our study is to identify predicting factors of long-term oncological outcomes following radical nephroureterectomy in the largest multicenter Taiwanese database.

## Materials and Methods

### Database Introduction

Ethics approvals were granted by the Internal Review Board of 15 participating hospitals, and data sharing agreements were required before commencing the multicenter cancer registry. In order to achieve consistent and accurate data registration, consensus meetings were undertaken to avoid any discrepancies. All patients in the database, Taiwan UTUC Collaboration Group, were waived from informed consent, and de-identified for privacy protection.

### Data Extraction

**Figure 1:**
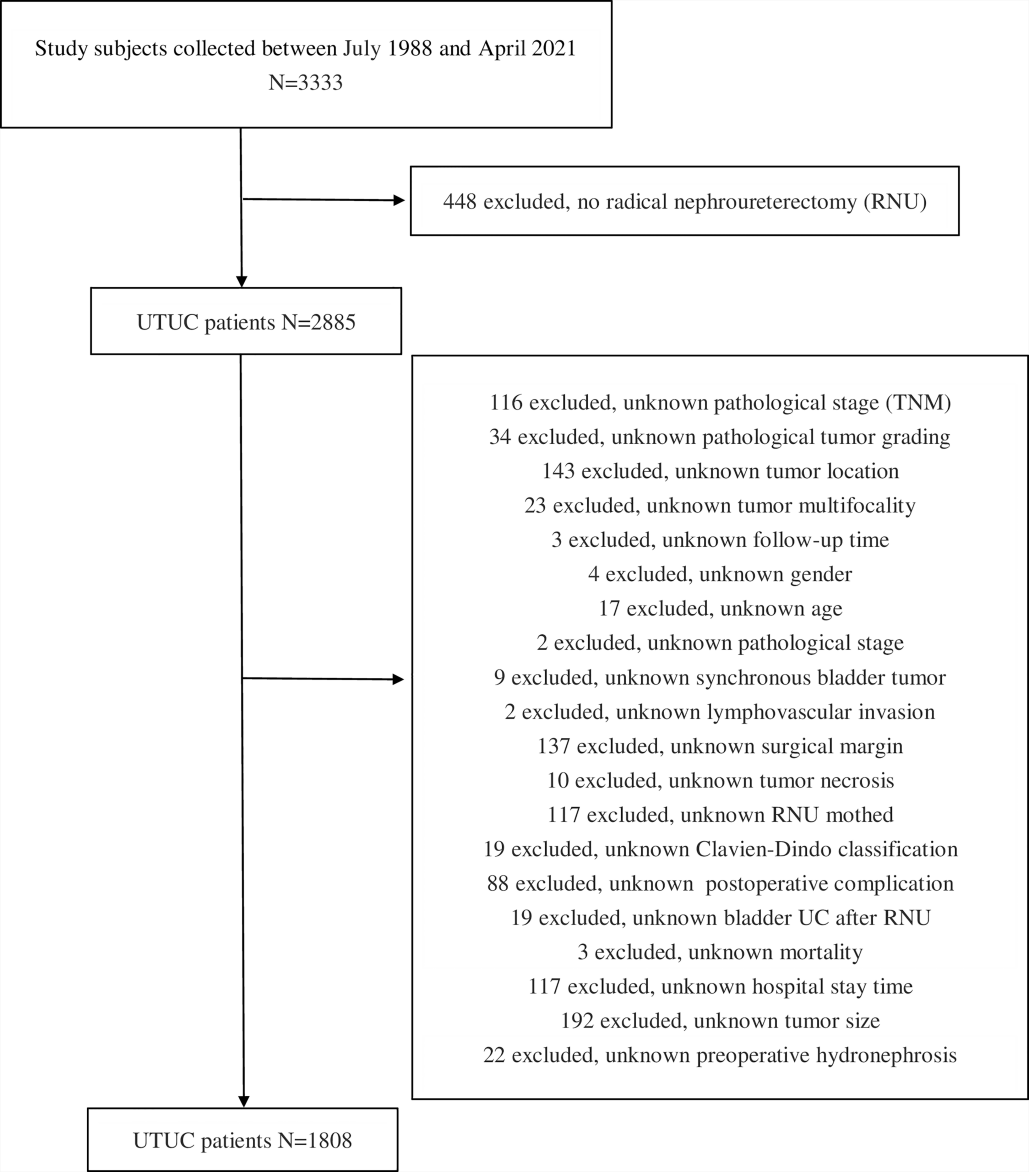
Study flowchart.

A total of 3,333 patients with UTUC from August 1988 to April 2021 inclusive were enrolled. Those undergoing RNU and bladder cuff excision were included in the current study. A variety of surgical approaches, including open and minimally invasive techniques, either transperitoneal or retroperitoneal, were presented. The exclusion criteria entailed 448 patients receiving kidney-sparing treatment and 1,077 patients who lack complete information, including basic characteristics, perioperative parameters, pathological results, and follow-up outcomes. On account of the retrospective nature of our large multicenter database, missing data could be expected, which was also inevitable in prospective multicenter studies. In order to maintain the robustness and completeness of our results, stringent exclusion criteria were applied. Incomplete data were prevented, and no imputation was undertaken for statistical analysis (**Figure 1**). No missing data was managed in all the data extracted. Patient demographics were recorded and postoperative complications were reported and graded using the Clavien-Dindo classification.

Tumor location and size were defined by evaluation of the specimen following RNU. Synchronous presence of two or more pathologically confirmed lesions at different sites (renal pelvicalyceal system or ureter) was designated as multifocality. Tumor size was calculated by summing the longest diameters of all tumors. Preoperative hydronephrosis was assessed utilizing the computed tomography (CT) or magnetic resonance imaging (MRI). Various cell types, carcinoma *in situ*, lymphovascular invasion (LVI), tumor necrosis, and surgical margins were reviewed by the urological pathologists. Histological grading was determined according to the 2004 World Health Organization grading system. Pathological staging was referenced according to the 2017 TNM staging system of the American Joint Committee on Cancer (AJCC). In addition, the presence and chronology of bladder UC were recorded.

### Survival Assessment

The primary endpoint of the study was to identify significant predicting factors of oncological outcomes, including overall survival (OS), cancer-specific survival (CSS), disease-free survival (DFS), and intravesical recurrence-free survival (IVRFS). The patients who died within 30 days after RNU or within the same hospital stay were censored at the time of mortality in the analysis of CSS. DFS was defined as time from RNU to either first local recurrence in the tumor bed, first lymph node or distant metastasis, or death from any cause. Recurrence and metastasis were assessed either radiologically or pathologically. Intravesical recurrence was coded with the presence of any subsequent histologically proven bladder UC during cystoscopy. All survival outcomes were evaluated with multivariate Cox proportional hazard models.

### Statistical Analysis

Continuous variables were tested for normality using Kolmogorov-Smirnov test. Continuous data were stratified into categories, and categorical data were reported as number and percentage of all patients. The Kaplan-Meier estimator was utilized to estimate the rates of prognostic outcomes, and the survival curves were compared using the stratified log-rank test. The Cox proportional hazard model was selected to assess the effect of clinicopathological parameters on the prognostic outcomes, alone and after adjusting for potential confounders. All statistical assessments were two-tailed and considered statistically significant as *p* < 0.05. Statistical analyses were carried out with IBM SPSS statistical software version 26.

## Results

### Patient, Tumors, and Surgical Approaches

The median follow-up for 1,808 patients undertaking RNU was 41.8 months; 482 (26.7%) patients experienced intravesical recurrence, 448 (24.8%) encountered disease recurrence outside of the bladder, 307 (17%) died of UTUC, and 583 (32.2%) died of any cause. The 5-year IVRFS, DFS, CSS, and OS probabilities were 66%, 72%, 81%, and 70%; the 10-year survival rates were 58%, 66%, 77%, and 51%, respectively. Patient demographics and pathological characteristics are shown in **Table 1**. The median age of diagnosis was 69 years, and 898 were equal to or more than 70 years old (49.7%). Gender predominance was female (57%); the most common sites of UTUC were renal pelvis (68%) and proximal ureter (22.7%). High-grade UTUC was diagnosed in 1,531 patients (84.7%); preoperative hydronephrosis presented in 1,094 patients (60.5%). Synchronous bladder UC was identified in 292 patients (16.2%). With regard to stage distribution, stage III predominated (29.4%) followed by stage I (24.9%) and stage II (18.6%). Interestingly, minimally invasive procedures accounted for 78.8% of all RNU surgeries, including 768 hand-assisted laparoscopic (42.5%), 494 laparoscopic (27.3%), 158 robot-assisted (8.7%), and 6 laparoendoscopic single site (LESS) (0.3%) approaches. The surgical margin was free in 1,732 patients (95.8%) but involved in 76 (4.2%) patients.

**Table 1:** Baseline demographics and clinicopathological characteristics.

Parameters	*N* (%)
Gender	
Men	777 (43.0)
Women	1,031 (57.0)
Age	
<70	910 (50.3)
≥70	898 (49.7)
Tumor location	
Renal pelvis	1,229 (68.0)
Proximal ureter	410 (22.7)
Middle ureter	252 (13.9)
Distal ureter	360 (19.9)
Bladder cuff	49 (2.7)
Tumor size	
Nonvisible	34 (1.9)
<1 cm	128 (7.1)
≥1 and <2 cm	356 (19.7)
≥2 and <3 cm	396 (21.9)
≥3 cm	894 (49.4)
Multifocality	
No	1182 (65.4)
Yes	626 (34.6)
Cell type	
Urothelial carcinoma (UC)	1,633 (90.3)
UC with variants	128 (7.1)
Squamous	1 (0.1)
Small cell	2 (0.1)
Others	44 (2.4)
Carcinoma *in situ* (CIS)	
No	1,497 (82.8)
Yes	311 (17.2)
Bladder UC	
No	1,392 (77.0)
Previous	124 (6.9)
Synchronous	292 (16.2)
Tumor grading	
Low grade	277 (15.3)
High grade	1,531 (84.7)
Lymphovascular invasion	
No	1,391 (76.9)
Yes	417 (23.1)
Surgical margin	
Free	1,732 (95.8)
Positive	76 (4.2)
Preoperative hydronephrosis	
No	714 (39.5)
Yes	1,094 (60.5)
Tumor necrosis	
No	1,522 (84.2)
Yes	286 (15.8)
Pathological stage	
0a/0is	334 (18.5)
I	450 (24.9)
II	337 (18.6)
III	531 (29.4)
IV	156 (8.6)
Pathological T stage	
pTis	26 (1.4)
pTa	308 (17.0)
pT1	453 (25.1)
pT2	346 (19.1)
pT3	590 (32.6)
pT4	85 (4.7)
Pathological N stage	
pN0	408 (22.6)
pN1	33 (1.8)
pN2	56 (3.1)
pNx	1,311 (72.5)
RNU techniques	
Open	382 (21.1)
Laparoscopic hand-assisted	768 (42.5)
Robot-assisted	158 (8.7)
Laparoscopic	494 (27.3)
LESS	6 (0.3)
RNU approaches	
Transperitoneal	951 (52.6)
Retroperitoneal	857 (47.4)
Clavien-Dindo classification	
No	1,093 (60.5)
Grade I	236 (13.1)
Grade II	365 (20.2)
Grade III	50 (2.8)
Grade IV	45 (2.5)
Grade V	19 (1.1)
Postoperative complication	
No	1528 (84.5)
Yes	280 (15.5)
ESRD	218 (12.1)
Ileus	45 (2.5)
Ventral hernia	33 (1.8)
Bladder UC following RNU	
No	1,326 (73.3)
Yes	482 (26.7)
Disease free	
No	1,360 (75.2)
Yes	448 (24.8)
UTUC-specific mortality	
No	1,501 (83.0)
Yes	307 (17.0)
Overall mortality	
No	1,225 (67.8)
Yes	583 (32.2)

RNU, radical nephroureterectomy; UTUC, upper tract urothelial carcinoma.

### Identification of Predicting Factors for OS

**Figure 2:**
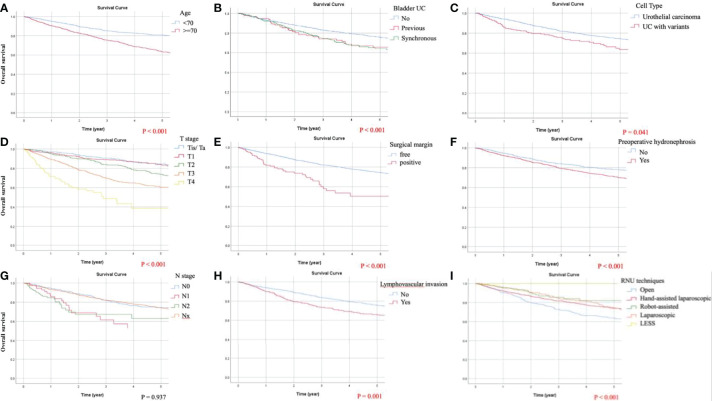
Kaplan-Meier curves of overall survival (OS) following adjustment for age, tumor size, preoperative hydronephrosis, distal ureteral or bladder cuff urothelial carcinoma (UC), multifocal UCs, previous or synchronous bladder UC, lymphovascular invasion (LVI), tumor necrosis, surgical margin, tumor grading, cell type, pathological T and N staging, and surgical approaches of radical nephroureterectomy (RNU). Significant predicting factors for OS included: **(A)** age, **(B)** chronological history of bladder UC, **(C)** cell type, **(D)** T stage, **(E)** surgical margin, **(F)** preoperative hydronephrosis, **(H)** LVI, and **(I)** RNU techniques. **(G)** N staging did not demonstrate significant influence on OS because the proportion of lymphadenectomy was limited in the present study.

In the univariate analysis of OS, the predictors demonstrating a *p*-value of <0.05 were taken into account in the subsequent multivariate analysis in which age, tumor size, preoperative hydronephrosis, distal ureteral or bladder cuff UC, multifocal UCs, previous or synchronous bladder UC, LVI, tumor necrosis, surgical margin, tumor grade, cell type, pathological T and N staging, and surgical approaches of RNU were included. Independent adverse predicting factors for OS were shown as follows: age ≧70, synchronous bladder UC, preoperative hydronephrosis, LVI, positive surgical margin, and pathological stages T2, T3, T4, N1, and N2. Adjusted Kaplan-Meier estimates of OS are demonstrated in **Figure 2**. Favorable predicting factors for OS were minimally invasive approaches, including laparoscopic (HR = 0.671), hand-assisted laparoscopic (HR 0.805), and robotic RNU (HR = 0.484).

### Identification of Predicting Factors for CSS

**Figure 3:**
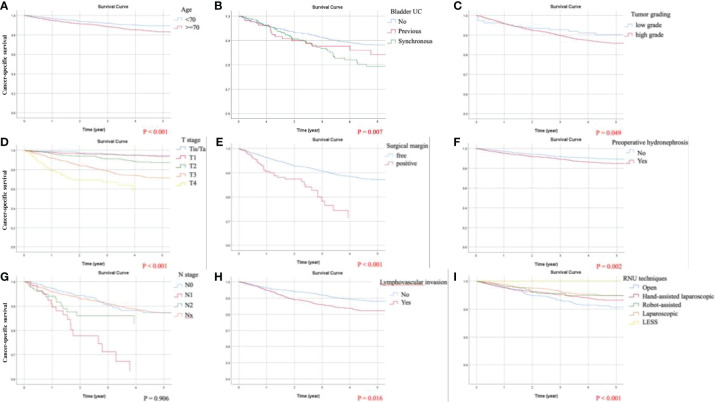
Kaplan-Meier curves of cancer-specific survival (CSS) following adjustment for age, tumor size, preoperative hydronephrosis, middle ureteral, distal ureteral or bladder cuff urothelial carcinoma (UC), multifocal UCs, previous or synchronous bladder UC, lymphovascular invasion (LVI), tumor necrosis, surgical margin, tumor grading, cell type, pathological T and N staging, and surgical approaches of radical nephroureterectomy (RNU). Significant predicting factors for CSS included: **(A)** age, **(B)** chronological history of bladder UC, **(C)** tumor grading, **(D)** T stage, **(E)** surgical margin, **(F)** preoperative hydronephrosis, **(H)** LVI, and **(I)** RNU techniques. **(G)** N staging did not demonstrate significant influence on CSS because the proportion of lymphadenectomy was limited in the present study.

By univariate analysis, worse CSS was associated with middle ureteral UC (HR = 1.372, *p* = 0.032); other statistically significant predictors for CSS included in the ensuing multivariate analysis were identical to those for OS. Independent adverse predicting factors for CSS were identified as follows: age ≧70, synchronous bladder UC, preoperative hydronephrosis, LVI, positive surgical margin, high-grade UC, and pathological stages T2, T3, T4, N1, and N2. Adjusted Kaplan-Meier estimates of CSS are shown in **Figure 3**. Merely one favorable predicting factor for CSS was laparoscopic RNU (HR = 0.551).

### Identification of Predicting Factors for DFS

**Figure 4:**
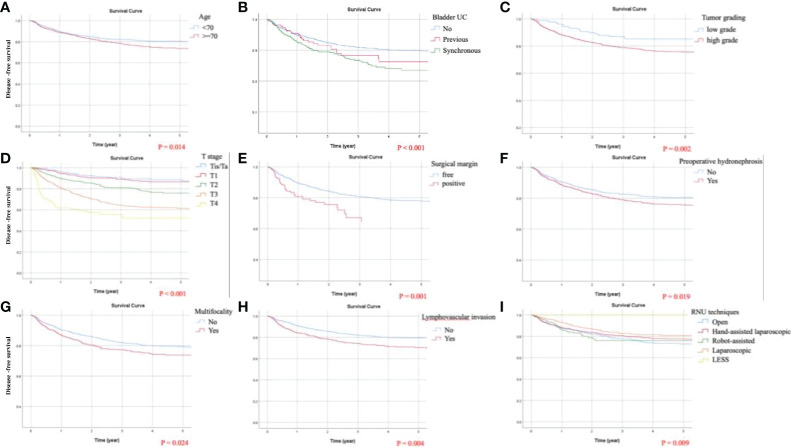
Kaplan-Meier curves of disease-free survival (DFS) following adjustment for age, tumor size, preoperative hydronephrosis, distal ureteral or bladder cuff urothelial carcinoma (UC), multifocal UCs, previous or synchronous bladder UC, lymphovascular invasion (LVI), tumor necrosis, surgical margin, tumor grading, cell type, pathological T and N staging, and surgical approaches of radical nephroureterectomy (RNU). Significant predicting factors for DFS included: **(A)** age, **(B)** chronological history of bladder UC, **(C)** tumor grading, **(D)** T stage, **(E)** surgical margin, **(F)** preoperative hydronephrosis, **(G)** multifocality, **(H)** LVI, and **(I)** RNU techniques.

By univariate analysis, except for robotic RNU, all statistically significant predictors for DFS included in the successive multivariate analysis were equivalent to those for OS. Independent adverse predicting factors for DFS were identified as follows: age ≧70, multifocal UC, synchronous bladder UC, preoperative hydronephrosis, LVI, positive surgical margin, high-grade UC, and pathological stages T2, T3, T4, N1, and N2. Adjusted Kaplan-Meier estimates of DFS are displayed in **Figure 4**. Only one favorable predicting factor for DFS was laparoscopic RNU (HR = 0.726).

### Identification of Predicting Factors for IVRFS

**Figure 5:**
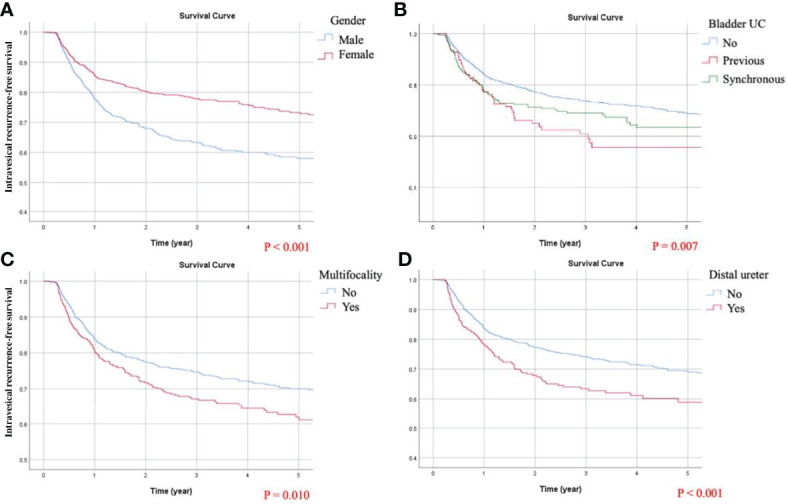
Kaplan-Meier curves of intravesical recurrence-free survival (IVRFS) following adjustment for gender, preoperative hydronephrosis, middle ureteral, distal ureteral or bladder cuff urothelial carcinoma (UC), multifocal UCs, previous or synchronous bladder UC, tumor grading, cell type, and pathological T staging. Significant predicting factors for IVRFS included: **(A)** gender, **(B)** chronological history of bladder UC, **(C)** multifocality, and **(D)** tumor position at the distal ureter.

In the univariate analysis of IVRFS, statistically significant predictors included gender, preoperative hydronephrosis, middle ureteral, distal ureteral or bladder cuff UC, multifocal UCs, previous or synchronous bladder UC, tumor grade, cell type, and pathological T staging. The following multivariate analysis highlighted that independent adverse predicting factors of BRFS were as below: distal ureteral UC, multifocal UCs, and previous and synchronous bladder UC. Adjusted Kaplan-Meier estimates of IVRFS are illustrated in **Figure 5**. Favorable predicting factors for BRFS were female gender (HR = 0.599) and pathological stage T4 (HR = 0.337).

All results of univariate and multivariate Cox regression analyses are depicted in **Tables 2**, **3**. Synchronous bladder UC was the dominant adverse predicting factor for all aspects of survival. Other independent predicting factors for OS, CSS, and DFS included age ≧70, presence of preoperative hydronephrosis, positive surgical margin, LVI, pathological T and N staging, and laparoscopic RNU.

**Table 2:** Comparative univariate and multivariate Cox regression analyses of overall survival (OS) and cancer-specific survival (CSS) in patients with UTUC.

	OS				CSS			
	Univariate		Multivariate		Univariate		Multivariate	
	HR (95% CI)	*p*	HR (95% CI)	*p*	HR (95% CI)	*p*	HR (95% CI)	*p*
Gender								
Female	0.90 (0.77–1.06)	0.226			0.93 (0.74–1.16)	0.500		
Age ≥70	2.13 (1.80–2.51)	<0.001^**^	2.16 (1.81–2.56)	<0.001^**^	1.63 (1.30–2.05)	<0.001^**^	1.59 (1.25–2.01)	<0.001^**^
Carcinoma *in situ*	1.11 (0.90–1.38)	0.323			0.987 (0.73–1.33)	0.930		
Tumor size								
Reference: <1 cm								
≥1 and <2 cm	0.94 (0.65–1.35)	0.726	0.84 (0.57–1.22)	0.358	0.91 (0.50–1.65)	0.750	0.63 (0.34–1.18)	0.148
≥2 and <3 cm	1.12 (0.79–1.59)	0.542	0.90 (0.62–1.31)	0.586	1.41 (0.81–2.46)	0.230	0.87 (0.48–1.56)	0.632
≥3 cm	1.69 (1.23–2.33)	0.001^**^	1.05 (0.74–1.50)	0.776	2.67 (1.601–4.45)	<0.001^**^	1.11 (0.64–1.94)	0.707
Tumor location								
Renal pelvis	0.98 (0.82–1.16)	0.808			1.02 (0.80–1.30)	0.865		
Proximal ureter	1.18 (0.98–1.43)	0.077			1.24 (0.96–1.60)	0.099		
Middle ureter	1.16 (0.93–1.45)	0.198			1.37 (1.03–1.83)	0.032^*^	1.08 (0.79–1.49)	0.626
Distal ureter	1.27 (1.04–1.54)	0.019^*^	1.14 (0.93–1.40)	0.219	1.45 (1.12–1.88)	0.005^**^	1.22 (0.92–1.63)	0.168
Bladder cuff	2.15 (1.47–3.14)	<0.001^**^	0.87 (0.57–1.32)	0.513	2.65 (1.65–4.27)	<0.001^**^	0.91 (0.53–1.54)	0.718
Multifocality	1.44 (1.22–1.70)	<0.001^**^	1.12 (0.94–1.33)	0.223	1.71 (1.37–2.14)	<0.001^**^	1.15 (0.90–1.48)	0.265
Preoperative hydronephrosis	1.56 (1.31–1.87)	<0.001^**^	1.43 (1.19–1.72)	<0.001^**^	1.70 (1.32–2.17)	<0.001^**^	1.52 (1.16–1.98)	0.002^**^
Lymphovascular invasion	2.50 (2.10–2.97)	<0.001^**^	1.38 (1.14–1.68)	0.001^**^	3.49 (2.79, 4.38)	<0.001^**^	1.36 (1.06–1.75)	0.016^*^
Positive surgical margin	4.35 (3.26–5.79)	<0.001^**^	1.93 (1.38–2.70)	<0.001^**^	6.03 (4.33, 8.41)	<0.001^**^	2.12 (1.42–3.16)	<0.001^**^
Tumor necrosis	1.62 (1.33–1.98)	<0.001^**^	1.10 (0.89–1.36)	0.392	1.92 (1.48, 2.49)	<0.001^**^	1.08 (0.82–1.43)	0.592
Tumor grading								
Low grade	1		1		1		1	
High grade	1.64 (1.29–2.10)	<0.001^**^	1.07 (0.82–1.40)	0.599	3.68 (2.26–6.01)	<0.001^**^	1.68 (1.00–2.81)	0.049^*^
Cell type								
Reference: urothelial carcinoma (UC)								
UC with variants	1.97 (1.55–2.51)	<0.001^**^	1.32 (1.01–1.71)	0.041^*^	2.60 (1.94–3.49)	<0.001^**^	1.37 (1.00–1.90)	0.054
Bladder UC								
Previous	1.18 (0.85–1.63)	0.314	1.37 (0.98–1.91)	0.065	1.05 (0.66–1.68)	0.842	1.34 (0.83–2.17)	0.238
Synchronous	1.55 (1.26–1.91)	<0.001^**^	1.50 (1.20–1.87)	<0.001^**^	1.69 (1.28–2.21)	<0.001^**^	1.52 (1.12–2.04)	0.007^**^
Pathological T stage								
Reference: Ta/Tis								
T1	1.16 (0.86–1.58)	0.328	1.16 (0.84–1.61)	0.359	1.35 (0.74–2.49)	0.330	1.20 (0.64–2.28)	0.570
T2	1.66 (1.23–2.24)	0.001^**^	1.40 (1.00–1.95)	0.048^*^	3.07 (1.75–5.40)	<0.001^**^	2.07 (1.12–3.79)	0.019^*^
T3	2.89 (2.21, 3.78)	<0.001^**^	2.20 (1.59–3.05)	<0.001^**^	7.94 (4.75–13.27)	<0.001^**^	4.70 (2.62–8.41)	<0.001^**^
T4	8.59 (6.04–12.22)	<0.001^**^	4.84 (3.15–7.45)	<0.001^**^	23.64 (13.29–42.04)	<0.001^**^	8.77 (4.47–17.20)	<0.001^**^
Pathological N stage								
Reference: N0								
N1	3.67 (2.27–5.93)	<0.001^**^	2.54 (1.55–4.17)	<0.001^**^	5.50 (3.27–9.25)	<0.001^**^	3.54 (2.05–6.13)	<0.001^**^
N2	3.05 (2.03–4.61)	<0.001^**^	1.87 (1.22–2.87)	0.004^**^	3.99 (2.42–6.57)	<0.001^**^	1.91 (1.13–3.23)	0.016^*^
N*x*	1.03 (0.83–1.27)	0.804	1.10 (0.88–1.37)	0.391	0.99 (0.74–1.33)	0.942	1.16 (0.86–1.56)	0.344
RNU techniques								
Reference: open								
Hand-assisted laparoscopic	0.79 (0.65–0.96)	0.017^*^	0.81 (0.66–0.99)	0.036^*^	0.71 (0.54–0.92)	0.009^**^	0.82 (0.62–1.09)	0.168
Robot-assisted	0.44 (0.28–0.70)	0.001^**^	0.48 (0.30–0.77)	0.002^**^	0.50 (0.29–0.85)	0.010^*^	0.60 (0.35–1.04)	0.067
Laparoscopic	0.62 (0.48–0.79)	<0.001^**^	0.67 (0.52–0.87)	0.002^**^	0.46 (0.33–0.65)	<0.001^**^	0.55 (0.39–0.79)	0.001^**^
LESS	0.24 (0.03–1.74)	0.160	0.15 (0.02–1.12)	0.064	0.48 (0.07–3.43)	0.462	0.21 (0.03–1.58)	0.128

* means “p < 0.05”; ** dictates “p < 0.01.

**Table 3:** Comparative univariate and multivariate analyses of disease-free survival (DFS) and intravesical recurrence-free survival (IVRFS) in patients with UTUC.

	DFS				IVRFS			
	Univariate		Multivariate		Univariate		Multivariate	
	HR (95% CI)	*p*	HR (95% CI)	*p*	HR (95% CI)	*p*	HR (95% CI)	*p*
Gender								
Female	0.91 (0.76–1.10)	0.314			0.55 (0.46–0.66)	<0.001^**^	0.60 (0.50–0.72)	<0.001^**^
Age ≥70	1.36 (1.13–1.64)	0.001^**^	1.27 (1.05–1.54)	0.014^*^	1.04 (0.87–1.24)	0.689		
CIS	1.06 (0.83–1.36)	0.645			1.22 (0.97–1.53)	0.092		
Tumor size								
Reference: <1 cm								
≥1 and <2 cm	0.94 (0.58–1.53)	0.816	0.74 (0.45–1.21)	0.225	0.95 (0.67–1.34)	0.751		
≥2 and <3 cm	1.40 (0.89–2.19)	0.145	0.99 (0.62–1.58)	0.956	0.88 (0.62–1.24)	0.452		
≥3 cm	2.63 (1.75–3.97)	<0.001^**^	1.35 (0.87–2.10)	0.184	0.99 (0.73–1.36)	0.972		
Tumor location								
Renal pelvis	1.12 (0.92–1.37)	0.274			0.90 (0.75–1.09)	0.269		
Proximal ureter	1.23 (1.00–1.52)	0.053			1.17 (0.95–1.44)	0.133		
Middle ureter	1.26 (0.98–1.62)	0.071			1.28 (1.00–1.63)	0.046^*^	1.11 (0.86–1.43)	0.416
Distal ureter	1.33 (1.07–1.65)	0.011^*^	1.23 (0.97–1.57)	0.084	1.70 (1.39–2.09)	<0.001^**^	1.49 (1.20– 1.85)	<0.001^**^
Bladder cuff	2.41 (1.58–3.67)	<0.001^**^	0.78 (0.49–1.25)	0.295	1.63 (1.02–2.61)	0.042^*^	1.07 (0.65– 1.76)	0.781
Multifocality	1.75 (1.45–2.11)	<0.001^**^	1.27 (1.03–1.55)	0.024^*^	1.57 (1.31–1.88)	<0.001^**^	1.30 (1.07–1.58)	0.010^*^
Preoperative hydronephrosis	1.37 (1.13–1.67)	0.002^**^	1.29 (1.04–1.59)	0.019^*^	1.29 (1.07–1.55)	0.008^**^	1.20 (0.99–1.46)	0.062
Lymphovascular invasion	3.26 (2.70–3.94)	<0.001^**^	1.37 (1.10–1.69)	0.004^**^	1.07 (0.86–1.34)	0.545		
Positive surgical margin	4.28 (3.14–5.83)	<0.001^**^	1.84 (1.29–2.64)	0.001^**^	0.88 (0.50–1.57)	0.668		
Tumor necrosis	1.85 (1.48–2.30)	<0.001^**^	1.04 (0.82–1.32)	0.754	0.96 (0.75–1.24)	0.767		
Tumor grading								
Low grade								
High grade	3.84 (2.56–5.74)	<0.001^**^	1.93 (1.26–2.94)	0.002^**^	0.80 (0.64–0.997)	0.047^*^	0.81 (0.63–1.03)	0.084
Cell type								
Reference: urothelial carcinoma (UC)								
UC with variants	2.14 (1.65–2.76)	<0.001^**^	1.24 (0.94–1.64)	0.128	0.65 (0.44–0.95)	0.027^*^	0.69 (0.47–1.02)	0.065
Bladder UC								
Previous	1.08 (0.74–1.58)	0.684	1.30 (0.88–1.93)	0.183	2.02 (1.50–2.71)	<0.001^**^	1.65 (1.22–2.23)	0.001^**^
Synchronous	1.76 (1.41–2.20)	<0.001^**^	1.62 (1.27–2.07)	<0.001^**^	1.68 (1.34–2.10)	<0.001^**^	1.33 (1.04–1.70)	0.022^*^
Pathological T stage								
Reference: Ta/Tis								
T1	1.37 (0.87–2.16)	0.176	1.16 (0.72–1.86)	0.551	1.06 (0.82–1.39)	0.647	1.17 (0.89–1.53)	0.269
T2	3.06 (2.01–4.67)	<0.001^**^	1.99 (1.27–3.14)	0.003^**^	1.13 (0.86–1.50)	0.378	1.18 (0.87–1.59)	0.291
T3	6.32 (4.29–9.30)	<0.001^**^	3.52 (2.27–5.46)	<0.001^**^	1.12 (0.87–1.45)	0.378	1.27 (0.96–1.69)	0.096
T4	16.77 (10.63– 26.45)	<0.001^**^	6.22 (3.65–10.60)	<0.001^**^	0.28 (0.10–0.77)	0.013^*^	0.34 (0.12–0.93)	0.035^*^
Pathological N stage								
Reference: N0								
N1	4.85 (3.03–7.76)	<0.001^**^	3.57 (2.19–5.83)	<0.001^**^	1.12 (0.55–2.30)	0.759		
N2	5.17 (3.50–7.65)	<0.001^**^	2.71 (1.79–4.09)	<0.001^**^	0.74 (0.36–1.52)	0.416		
N*x*	1.04 (0.82–1.32)	0.759	1.23 (0.96–1.58)	0.110	0.97 (0.78–1.21)	0.802		
RNU techniques								
Reference: open								
Hand-assisted laparoscopic	0.76 (0.61–0.96)	0.020^*^	0.98 (0.77–1.25)	0.875	1.15 (0.90–1.48)	0.258		
Robot-assisted	0.80 (0.55–1.17)	0.249	1.01 (0.68–1.50)	0.953	1.11 (0.75–1.64)	0.611		
Laparoscopic	0.59 (0.45–0.78)	<0.001^**^	0.73 (0.55–0.96)	0.027^*^	1.17 (0.89–1.54)	0.250		
LESS	0.34 (0.05–2.46)	0.286	0.20 (0.03–1.46)	0.111	0.83 (0.21–3.39)	0.800		

* means “p < 0.05”; ** dictates “p < 0.01.

## Discussion

Despite the high prevalence of UTUC in Taiwan, patient demographics and perioperative data on a domestic basis are lacking. In order to better understand the behavior of UTUC in one of the endemic regions, a multicenter Taiwan UTUC Collaboration Group was established by 15 participating hospitals to collect detailed clinical information. In our large multicenter cohort of 1,808 patients receiving RNU, female predominance was observed, which was corresponding to previous hospital-based results (8, 10) as well as the crude incidence rate from the Taiwan Cancer Registry Annual Report. Different gender distributions were ascertained as compared with the reports from Western (11, 12) and other Asian countries (13, 14). Similar to previous studies (15, 16), no gender difference could be demonstrated in OS or CSS. Nevertheless, Huang et al. (10) highlighted that females had better OS and CSS in nonmuscle invasive UTUC; similarly, better IVRFS was exhibited in our female patients.

Approximately 8% to 13% of patients with UTUC present with synchronous bladder UC (17). In the French national UTUC database, 9.4% of the enrolled 662 patients showed synchronous bladder UC; 16.2% was reported in our study. It is noteworthy that synchronous bladder UC was an independently adverse predicting factor for OS, CSS, DFS, and IVRFS. Likewise, Mullerad et al. (18) maintained that a history of superficial or muscle-invasive bladder cancer was independently associated with CSS and IVRFS. Given that their survival analysis might be skewed by muscle-invasive bladder UC, Pignot et al. (17) focused on the influence of previous or synchronous superficial bladder UC unambiguously. As expected, the history of superficial bladder UC is a well-known predictor of intravesical recurrence (IVR), but they failed to reveal a prognostic effect on survival. Interestingly, as chronology was taken into consideration, the survival differences between previous and synchronous bladder UC were significantly manifested in the current study. Moreover, a previous bladder UC was again proven as a predicting factor for IVR.

In spite of a similar histologic appearance, distinct epidemiologic and clinicopathologic differences have been identified between UTUC and bladder UC (19, 20). Nevertheless, Doeveran et al. (21) conducted a systematic review to underline that UTUC and paired bladder UC (synchronous or metachronous) were likely clonally related. Later, they performed targeted genomic sequencing to support the hypothesis that metachronous bladder UCs following RNU were predominantly clonally derived recurrences (22). Furthermore, Petros et al. (23) indicated that, regardless of chronologic development or anatomic origin, most metachronous tumors maintained molecular subtype membership of the initial tumor. Most relevantly, the whole transcriptome RNA sequencing demonstrated luminal-like gene expression in same-patient samples of UTUC and synchronous bladder UC. When examining gene expression profiles of basal/luminal immunohistochemical markers, Sikic et al. (24) reported the luminal-like (CD20+/CK5−) subtype to be associated with worse cancer-specific survival. Given that most tumor cells of UTUC and paired bladder UC shared identical clonality, either UTUC metastasis to the bladder or bladder cancer metastasis to the upper tract, it is plausible to speculate that intraluminal cancer seeding may be a pivotal mechanism for drop or retrograde metastasis. Certainly, synchronous upper tract and bladder UCs express in a similar fashion and an aggressive clinical behavior of such disease entity may be expected.

Since preoperative hydronephrosis was regarded as a controversial risk factor, Tian et al. (25) conducted a thorough systematic review and meta-analysis to clarify its role in the prognosis of UTUC. They suggested that preoperative hydronephrosis was significantly associated with poor survival. Similarly, the latest two-center Japan study (26) depicted that preoperative hydronephrosis was an independent predictor of shorter recurrent-free survival. To the best of our knowledge, the present study is the largest one investigating the relationship between preoperative hydronephrosis and oncological outcomes. With adjustment of potential confounding factors, it was independently associated with OS, CSS, and DFS. One possible mechanism to shed light on our finding is that the presence of preoperative hydronephrosis may mostly be attributed to luminal obstruction caused by ureteral tumors. In the present study, more than 90% of patients presenting with preoperative hydronephrosis had ureteral tumors. Although the prognostic role of primary tumor location remains contentious, Yu et al. (9) pointed out that ureteral UC was a significantly adverse predicting factor for OS, CSS, DFS, and IVRFS, in comparison with renal pelvic UC. Moreover, a thin-walled structure of the ureter with an extensive anastomosing network of arterial supply and venous and lymphatic drainage may be one of the mechanisms which promote cancer spreading and poorer prognosis. Another explanation is that some renal pelvic tumors may block the ureteropelvic junction and increase intrarenal pressure that impede flow of lymphatics and vasculature, which might induce increased cancer seeding (27).

A systematic review of European Association of Urology (28) suggested that the oncological outcomes of open RNU may be better than those of laparoscopic RNU as bladder cuff was excised laparoscopically and in locally advanced high-risk UTUC. Despite better perioperative outcomes utilizing the laparoscopic approach, its oncological safety continues to be debatable. Even though some propensity-score matching analyses were presented, no consistent conclusion can be drawn (29, 30). In the most recent meta-analysis comparing laparoscopic versus open RNU, Piszczek et al. (3) found comparable oncological outcomes in UTUC patients, even in locally advanced disease. Intriguingly, our multivariate analysis showed better OS, CSS, and DFS for the laparoscopic surgical approach. It partly can be explained by the high incidence of UTUC in Taiwan, which contribute to high surgical volume in Taiwanese regional hospitals and medical centers. Notwithstanding there was no census regarding the number of RNU per year recognized as high surgical volume, regional variations were clearly described in the reviewed literature. In the States, from the National Cancer Database, Sui et al. (31) defined high-volume hospitals as more than 6 RNU performed each year. The results of their multivariate analyses accorded with our assumption, which indicated that performance of RNU at high-volume hospitals was associated with better long-term survival. In Japan, Sugihara et al. (32) depicted less than 20 procedures per year as low-volume institutes. They found that minimally invasive RNU was more likely to be offered at high-volume hospitals. In our series, with a cutoff level of 20 minimally invasive RNU each year, higher hospital volume (≧20) was significantly associated with better OS. All these results corroborate our explanation that surgical volume may be a pivotal predicting factor in survival following RNU.

Of note, a high proportion (72.7%) of minimally invasive approaches was evident in our contemporary cohort. Whereas one theory attributed recurrence to carbon dioxide insufflation and pneumoperitoneum, neither port site metastasis nor peritoneal dissemination was registered in the present study. Another possible mechanism explaining better survival following laparoscopic RNU is delicate manipulation of the upper tract with meticulous prevention of urine spillage. Early ureteral clipping to reduce drop metastasis and prompt urine drainage to avoid cancer seeding are of paramount importance in our surgical training and routine practice of RNU. Additionally, when observing the trend of different RNU approaches within decades, the numbers of minimally invasive RNU have been increasing since 2000. Between 2006 and 2015, the most common approach was hand-assisted laparoscopic RNU in Taiwan. It can be alluded that the hand-assisted laparoscopic procedure might accelerate the transition of open to laparoscopic RNU. Not only could it preserve the conventional open method of bladder cuff excision, but also it assisted in the development of laparoscopic ureteric, perihilar, and perirenal dissection. After such transitional period, the proportion of laparoscopic RNU became the largest between 2016 and 2021. Simultaneously, the number of robotic RNU has been increasing since 2011. Undoubtfully, selection bias favoring the laparoscopic approach was commonly observed in a myriad of studies (28). In our series, with reference to T4 tumors, 36 (9.4%) patients were in the open RNU group and 15 (3.0%) in the laparoscopic group. Regarding T3 tumors, the numbers of patients were similar in both open (126, 33%) and laparoscopic (165, 33.4%) approaches. Undeniably, as UTUC invaded adjacent organs, surgeons still preferred open surgery for T4 tumors. Nevertheless, our registry data revealed that minimally invasive operations were yet undertaken in patients with locally advanced or even nodal diseases. With accumulated surgical experience of RNU, regardless of open or minimally invasive access, Taiwanese urologists became accustomed to various pathological circumstances and delivered better quality of surgical oncology practice, thereby explaining better survival outcomes for the laparoscopic approach.

Several limitations of the present study merit discussion. Firstly, the data were retrospectively recruited and analyzed. On account of multi-institutional collaborations, these operations were performed by various surgeons at each institution and the surgical approach, especially pertaining to the management of the distal ureteral cuff, was decided at individual’s discretion. Nevertheless, potential confounding factors were adjusted by multivariate Cox regression analyses to identify independently significant predictors. Furthermore, the multi-institutional study included a wider range of population groups, increasing the generalizability of the results. Secondly, lymph node yields and precise nodal status were lacking. Given there was no substantial evidence of therapeutic effect and standardized template of regional lymphadenectomy, it was merely provided in UTUC patients with suspiciously nodal disease. Thirdly, centralized pathological and radiological reviews were not conducted. To mitigate the influence of intra- and interobserver variability, we utilized a standardized format that was based on the principles of pathology management for urothelial cancer in the NCCN guidelines and the AJCC TNM staging system, to ensure accordance of interpretation. Additionally, neoadjuvant or adjuvant systemic therapy was not depicted in the present study. The patients receiving systemic therapy accounted for a fairly small portion of our database. Even though these patients were excluded from the present cohort, our outcomes remained unchanged.

Another limitation needs to be addressed: the pathological staging of synchronous bladder UC was not registered in our database. With regard to the bladder disease, complexity would be expected and more sophisticated variables were required, such as tumor location (trigone, ureteral orifices or other parts of the urinary bladder), intravesical chemotherapy or Bacillus Calmette-Guérin induction or maintenance, subsequent treatment modalities (systemic chemotherapy, chemoradiation or radical cystectomy), and recurrent disease status. Owing to limited human resources, after discussion within our consensus conferences, details of synchronous bladder UC were reduced to the presence or not of the disease. Nevertheless, in our experiences, most of them were nonmuscle invasive UC of the urinary bladder, because merely 33 patients in our cohort received systemic chemotherapy for bladder cancer. Only 2 of them underwent neoadjuvant chemotherapy and hence it might be speculated that the number of simultaneous radical cystectomy was extremely low in our database. It is plausible that most patients with synchronous bladder UC were treated by transurethral endoscopic resection.

Undoubtedly, the retrospective nature of the multi-institutional study introduced hospital variations and selection bias. However, a single-institution experience could hardly represent the clinical behavior of UTUC in Taiwan. Notwithstanding the rarity of this disease around the world, the long-term observations from our multicenter effort may contribute to improved prognostic prediction and surgical treatment advances. Following statistical control of confounding factors, several significantly beneficial and adverse predictors were identified. Further prospective well-designed researches are warranted to validate our findings and elucidate the underlying mechanism. In light of the real-world context, we believe this multi-institutional collaboration may be a considerable help in medical progress of treating UTUC.

## Conclusion

This multi-institutional collaborative study in Taiwan recognized synchronous UC in the urinary bladder as a harbinger of poor prognosis for patients with UTUC. In addition, the presence of preoperative hydronephrosis was corroborated as an adverse prognostic factor for UTUC. Interestingly, our multivariate analysis suggested laparoscopic RNU might provide better oncological control. Further randomized controlled trials are warranted to validate our finding.

## Data Availability Statement

The raw data supporting the conclusions of this article will be made available by the authors, without undue reservation.

## Ethics Statement

The studies involving human participants were reviewed and approved by Kaohsiung Veterans General Hospital (IRB No.: VGHKS14-CT3-06). Written informed consent for participation was not required for this study in accordance with the national legislation and the institutional requirements.

## Author Contributions

C-HCha, C-PH, W-JW, and C-CL contributed to conception and design of the study. C-HChe and C-YH organized the database. CC-L, C-ChiY, and C-YT performed the statistical analysis. I-HC wrote the first draft of the manuscript. W-CW, J-ST, W-RL, Y-HJ, Y-KL, Y-CJ, I-SC, TH, AC, Y-TC, J-SC, B-JC, Y-CT, WL, C-CW, J-TL, and C-CheY wrote sections of the manuscript. All authors contributed to manuscript revision and read and approved the submitted version.

## Conflict of Interest

The authors declare that the research was conducted in the absence of any commercial or financial relationships that could be construed as a potential conflict of interest.

## Publisher’s Note

All claims expressed in this article are solely those of the authors and do not necessarily represent those of their affiliated organizations, or those of the publisher, the editors and the reviewers. Any product that may be evaluated in this article, or claim that may be made by its manufacturer, is not guaranteed or endorsed by the publisher.
